# Joint impact of the number of comorbidities and the COVID-19 pandemic on the concordance of antibiotic prescriptions with clinical guidelines, in the community

**DOI:** 10.1186/s12879-026-13948-6

**Published:** 2026-07-08

**Authors:** Philip Verret, Élise Fortin, Caroline Sirois, Caroline Quach, Marc Simard, Nadine Magali-Ufitinema, Chantal Sauvageau

**Affiliations:** 1 Institut national de santé publique du Québec (INSPQ), Québec, Québec Canada; 2 Department of Social and Preventive Medicine, Faculty of Medicine, Laval University, Québec, Québec Canada; 3 Department of Microbiology, Infectiology and Immunology, Faculty of Medicine, University of Montreal, Québec, Montréal, Canada; 4 Faculty of Pharmacy, Laval University, Québec, Québec Canada; 5 Direction de la vigie et des maladies infectieuses, Direction générale de la protection de la santé publique, Ministère de la Santé et des Services sociaux, Québec, Québec Canada; 6 Research Center of CHU de Québec-Université Laval, Québec, Québec Canada

**Keywords:** Antimicrobial resistance, Antibiotic prescribing, Appropriateness, Comorbidities, COVID-19, Respiratory infections, Urinary tract infections

## Abstract

**Background:**

This study aimed to describe community antibiotic use for respiratory and urinary tract infections (UTIs), in Quebec, from 2018 to 2022 and to assess the joint effect of comorbidities and the COVID-19 pandemic on prescription concordance with provincial guidelines.

**Methods:**

A retrospective analysis was conducted using medical and pharmaceutical claims data from 2018 to 2022 for individuals insured by the public health and drug plans. Data were sourced from the Quebec Integrated Chronic Disease Surveillance System (QICDSS), linking five administrative databases. The primary outcome was the proportion of prescriptions concordant with the provincial guidelines for the most frequent infections in children and adults. Prescriptions dispensed within two days of a medical diagnosis for eligible infections were included. Robust Poisson regressions assessed the joint impact of number of comorbidities and pandemic period on concordance.

**Results:**

In children, over 90% of antibiotic prescriptions were concordant with guidelines. Lower concordance was observed for pneumonias and pharyngitis among children with comorbidities. In adults, concordance varied by infection type and declined with increasing comorbidities. Concordance improved during the pandemic and after for UTIs and rhinosinusitis, but decreased for pneumonias. No significant interactions were found between comorbidities and pandemic periods, except for bronchitis with chronic obstructive pulmonary disease (COPD).

**Conclusion:**

In Quebec, comorbidities were associated with decreased adherence to clinical guidelines for antibiotic prescribing, particularly among adults. The COVID-19 pandemic had minimal influence on concordance.

**Clinical trial number:**

Not applicable.

**Supplementary Information:**

The online version contains supplementary material available at https://doi.org/10.1186/s12879-026-13948-6.

## Introduction/background

Antimicrobial resistance (AMR) is the ability of microorganisms to withstand treatment with antimicrobial drugs, making infections harder to treat. It is a rising global threat to public health according to many health authorities [1–6]. The lack of new antibiotics and the increase of multidrug-resistant bacteria make treatments more difficult or even impossible, resulting in severe complications for the patient, potentially leading to death [7]. Appropriate antibiotic use does not eliminate this phenomenon, but improper use and overuse worsen it [8]. The situation is even more troubling for people with comorbidities, who are known to use more antibiotics [9–11]. They are also more vulnerable due to their pre-existing conditions, which increase their risk of bacterial infections and exposure to potentially inappropriate prescriptions, amplifying the issues of antimicrobial resistance [12]. Moreover, the pandemic has brought many changes to the healthcare system, which may have disproportionately impacted people with chronic medical conditions [13–15]. Conversely, antimicrobial use declined in Québec during the pandemic; however, the reduction was less pronounced among patients with at least three comorbidities. This pattern may suggest relatively more stable access to healthcare for these patients, although there is no strong evidence to conclusively support this interpretation [16].

To curtail antibiotic resistance, global surveillance systems and evidence-based guidelines to monitor and optimize antimicrobial use (AMU) are important. At the international level, the World Health Organization (WHO) leads global initiatives through the Global Antimicrobial Resistance (AMR) and Use Surveillance System (GLASS) [17], and many developed countries have their own surveillance program, providing crucial data to guide antibiotic stewardship and combat resistance [2, 6, 18, 19]. In Québec, Canada, there is no integrated surveillance of AMR and AMU. Our project aimed to analyze the concordance of antibiotic prescriptions filled in Quebec community pharmacies with provincial guidelines, focusing on the combined effects of the number of comorbidities and of the COVID-19 pandemic during the period from April 2018 to March 2022.

## Methods

### Study design and population

This project was a retrospective population-based study analysis of all antibiotic prescriptions filled in community-based pharmacies between April 1st, 2018, and March 31st, 2022, for an infection for which provincial treatment guidelines exist (otitis media, respiratory and urinary infections). The study population included all individuals insured by the public health and drug insurance plans for at least one day during this period. In 2021–2022, over 8.3 million people were insured under the public health insurance plan, and more than 3.8 million (45.8%) under public drug insurance plan [20]. Residents in the province of Quebec are required to have prescription drug insurance through a public or private plan. Individuals 65 years and older receive automatic coverage under the public drug insurance plan, whereas for those under 65 years of age, coverage is restricted to people without private insurance, such as self-employed individuals or individuals on social assistance [21, 22]. The public drug insurance plan covers over 90% of individuals 65 years and older, but only one-third of those under 65 [23]. People with lower socio-economic status are overrepresented in the public drug insurance plan, as those under 65 insured by this plan generally have a lower socio-economic status compared to those with private insurance [23].

### Data sources and variables

The data used in this study were obtained from the Quebec Integrated Chronic Disease Surveillance System (QICDSS) [24], which links information from five health administrative databases: (1) health insurance registry (*Fichier d’inscription des personnes assurées*: FIPA), (2) physician claims (information source for infection diagnoses), (3) pharmaceutical services (information source for antibiotic prescriptions), (4) hospital inpatient and day surgery (*Maintenance et exploitation des données pour l’étude de la clientèle hospitalière*: MED-ECHO) and (5) vital statistics death. Data linking was based on QICDSS’ unique identifier, individuals’ encrypted health insurance number [24, 25].

#### Infections under study in children and adults

Antibiotic prescriptions included in the study were those filled in community-based pharmacies two days or less after a physician claim for an infection for which a provincial guideline exists [26]. In children (0–17 years old), these infections are otitis media, pharyngitis, pneumonias and rhinosinusitis. In adults, studied infections are cystitis and pyelonephritis, bronchitis with chronic obstructive pulmonary disease (COPD) for adults aged 35 years old and older, pharyngitis, rhinosinusitis and pneumonias [26]. We also identified prescriptions issued for upper respiratory tract infections (URTIs), for which no antibiotic is recommended. Diagnostic codes are listed in Appendix 1. Infections were identified using international classification of diseases codes (ICD-9 and ICD-10) in physician claims. Any prescriptions linked to two or more distinct infection diagnoses on the same day (< 0.5% of prescriptions) were excluded as it was impossible to determine for which infection the prescription was delivered.

Recurrent infections, defined as an infection of the same type occurring within 90 days of an initial diagnosis for the same individual, were excluded from the analyses. This criterion ensures that more severe cases, where initial treatment may have failed and where deviations from guidelines could be justified, were not included.

#### Antibiotics and concordance with guidelines

Only systemic antibiotics from the Anatomical Therapeutic Chemical classification system (ATC: J01 and P01AB01) were included in the analysis [27]. Prescriptions were classified according to their adherence to the 2016–2017 clinical guidelines, which are summarized in Appendix 2 (concordant, non-concordant or a combination of concordant and non concordant antibiotics). To be considered concordant, the prescribed antibiotic had to be among agents recommended for the diagnosed infection. When two antibiotic prescriptions were dispensed the same day for the same infection, they were considered as an antibiotic combination. For this combination to be concordant, every antibiotic had to be amongst the recommended options for the infection. For adults and for oral use only, the total antibiotic dosage dispensed had to fall within the recommended limits (duration of treatment was not available); pediatric dosages and parenteral doses were excluded from dosage analyses due to lack of information on patient weight and quantity of antibiotic (mg/mL), respectively. Administration route was determined using form code. Antibiotic prescriptions for URTIs were all considered potentially inappropriate given their usual viral etiology [28]. Diagnostic codes are not mandatory in the physician claims, so while the concordance proportion can be measured when a diagnosis is found and a prescription is delivered, numbers are a large underestimation of the reality. Along the same lines, non-prescription cannot be documented with this approach.

#### Comorbidities and COVID-19 pandemic periods

Comorbidities were identified using diagnostic codes in the hospitalizations and physician claims data and applying validated algorithms developed specifically for the QICDSS to compute the combined comorbidity index of Charlson and Elixhauser [23, 29]. Algorithms were the same for adults and children. Adults were grouped into categories of 0, 1, 2, and 3 or more comorbidities, while children were classified based on the presence or absence of comorbidities. These categories were established to reflect differences in antibiotic use rates associated with the number of comorbidities and to minimize categories with few observations.

The temporal evolution of antibiotic prescriptions was analyzed in three pandemic periods, defined in a previous study: the pre-pandemic period (April 2018 to March 2020), the pandemic period (April 2020 to July 2021), and the post-pandemic period (August 2021 to March 2022) [16]. These periods were defined based on prescription trends and the evolving public health situation in Quebec [16].

#### Covariates

Information on sex, age (0–1, 2–4, 5–11, 12–17,18–29, 30–49, 50–64, 65–74, and 75 + years old), geographic area (urban or rural), and indexes of social and material deprivation was also collected for all prescriptions. These indices are assigned at the postal‑code level and do not reflect individual‑level deprivation; however, they represent the best available approach to incorporating socio‑economic information into our administrative databases [30]. Prescriptions with missing data for covariates were excluded from analyses, except for deprivation indexes, for which an “unknown” category was created due to the larger number of missing values.

### Statistical analyses

Proportions of antibiotic prescriptions concordant with clinical guidelines were measured, stratified according to the number of comorbidities and pandemic period for each infection type. Children and adults were analyzed separately.

Robust Poisson regressions (using Generalized Estimating Equations with an exchangeable correlation structure, and where the clustering unit is the individual) were conducted to measure the joint impact of the number of comorbidities and the COVID-19 pandemic on the concordance of antibiotic prescriptions. Interaction terms were used to assess the presence of interaction between comorbidities and pandemic periods. This method also enabled us to evaluate the basic effect of each independent variable individually. We used a Type III test to assess the significance of interaction terms via a chi-square test. When the p-value was not significant (α = 0,05), the model without interaction terms was preferred. Regression analyses were all adjusted for sex, age, geographic area and indexes of social and material deprivation. The only exception was for pneumonias in children, for which the fully adjusted model predicted proportions above 1; for this model, we excluded both deprivation indexes (with little impact on the quasi-likelihood under the independence model criterion [QIC]) and we regrouped children aged five to seventeen years old together. Sensitivity analyses were performed to examine the impact of antibiotic combinations. In the main analysis, combinations that included recommended and non-recommended antibiotics were classified as non-concordant. In the sensitivity analysis, these treatments were considered concordant.

## Results

### Concordance of antibiotic prescriptions with provincial guidelines in children

Under the public drug insurance plan, children received 89,771 antibiotic prescriptions for otitis, pharyngitis, pneumonias, rhinosinusitis, or URTIs between April 2018 and March 2022 (Table 1). Antibiotics prescribed for studied infections were generally among the recommended options, with compliance rates reaching 90% and more across all infection types or groups of comorbidities. Amoxicillin was the most prescribed antibiotic in all infection types except URTIs (data not shown). The 13,554 prescriptions associated with URTIs (likely viral) were considered non-concordant. Concordance proportions by year for each comorbidity group and for each infection in children is presented in Appendix 3 (Fig. 1).

**Table 1:** Concordance of antibiotic prescriptions with provincial guidelines by number of comorbidities, by infection, in children**

Infection	Number of comorbidities	Prescriptions	Concordant	Concordance Concordant + other*	Non-Concordant
		(N)			
Otitis	0	37 981	99.1%	0.2%	0.7%
	1+	5 998	98.7%	0.3%	1.0%
	Total	43 979	99.0%	0.2%	0.8%
Pharyngitis	0	16 945	92.7%	0.3%	7.0%
	1+	2 933	90.8%	0.3%	8.9%
	Total	19 878	92.4%	0.3%	7.3%
Pneumonias	0	6 795	98.6%	0.7%	0.7%
	1+	2 090	97.4%	1.2%	1.4%
	Total	8 885	98.3%	0.8%	0.8%
Rhinosinusitis	0	2 765	94.0%	0.4%	5.6%
	1+	710	93.0%	1.4%	5.6%
	Total	3 475	93.8%	0.3%	5.9%
URTI	0	11 641	0.0%	0.0%	100.0%
	1+	1 913	0.0%	0.0%	100.0%
	Total	13 554	0.0%	0.0%	100.0%

Note: URTI = Upper respiratory tract infection, Provincial guidelines from 2016–2017, April 2018 to March 2022

*Concordant + other: when at least one recommended antibiotic is included in a combination of antibiotics, meaning more than one antibiotic is prescribed on the same day for the same infection

**In order to protect the identity of individuals in cells with small numbers, all numbers for an infection with at least one small number are rounded to the nearest multiple of 5. Therefore, the totals may slightly differ from one variable to another

As URTIs were considered viral infections, the 13,554 prescriptions associated were considered non-concordant

The analysis of the combined effects of the number of comorbidities and the COVID-19 pandemic on antibiotic concordance revealed no statistically significant interaction between the two variables, for any type of infection in children (Table 2). Adjusting for social and material deprivation indexes had little impact on our results (Table 2). We observed no statistically significant interaction between the two variables and oriented our analysis to the distinct effect of each variable for each infection. The presence of comorbidities showed a small but statistically significant decrease in concordance for children with comorbidities diagnosed with pneumonias (0.988, 95% CI: 0.980–0.996) and pharyngitis (0.982, 0.970–0.994) as shown in Table 2. Furthermore, we observed slightly less concordant prescriptions in children with otitis during the pandemic period (0.995, 0.991–0.999) and children with pharyngitis during the post-pandemic period (0.983, 0.967–0.998). The magnitude of all statistically significant effects was always small. Sensitivity analyses did not change conclusions, as shown in Table S1 in Appendix 4.

**Table 2:** Multivariate analysis of the impact of comorbidities and pandemic periods on concordance, in children

Children	Pneumonias**		Pharyngitis			Rhinosinusitis		Otitis	
Variable	Crude RR (95% CI)	Adjusted RR* (95% CI)	Crude RR (95% CI)		Adjusted RR* (95% CI)	Crude RR (95% CI)	Adjusted RR* (95% CI)	Crude RR (95% CI)	Adjusted RR* (95% CI)
**Period**									
Pre-pandemic period (April 2018 to March 2020)	Ref.	Ref.	Ref.		Ref.	Ref.	Ref.	Ref.	Ref.
Pandemic period (April 2020 to July 2021)	0.987 (0.969–1.005)	0.984 (0.966–1.002)	1.001 (0.987–1.015)		1.007 (0.993–1.022)	0.969 (0.927–1.013)	0.966 (0.924–1.010)	0.995 (0.991–0.999)	0.995 (0.991–0.999)
Post-pandemic period (August 2021 to March 2022)	1.001 (0.992–1.010)	0.997 (0.989–1.006)	0.978 (0.963–0.995)		0.983 (0.967–0.998)	0.994 (0.967–1.023)	0.992 (0.963–1.021)	0.998 (0.995–1.001)	0.998 (0.995–1.001)
**Number of comorbidities**									
0	Ref.	Ref.	Ref.		Ref.	Ref.	Ref.	Ref.	Ref.
1+	0.989 (0.980–0.996)	0.988 (0.980–0.996)	0.980 (0.968–0.993)		0.982 (0.970–0.994)	0.989 (0.968–1.023)	0.990 (0.969–1.023)	0.997 (0.994-1.000)	0.997 (0.994-1.000)

Note: CI = Confidence interval, RR = Relative risk, Ref = Reference category, Provincial guidelines from 2016–2017, April 2018 to March 2022, Only antibiotic choice is included in the concordance definition

*The adjusted models are adjusted for sex, age group, area of residence, social and material deprivation indexes

** In order to protect the identity of individuals in cells with small numbers, for pneumonias in children, the model was not adjusted for the social and material deprivation indexes and the 12–17 years old age group was included in the 5–11 years old age group

### Concordance of antibiotic prescriptions with provincial guidelines in adults

Under the public drug insurance plan, we retrieved a total of 374,571 prescriptions for adults between April 2018 and March 2022 for bronchitis with COPD, pharyngitis, pneumonias, rhinosinusitis, cystitis, pyelonephritis, or URTIs (Table 3). The agent of choice for respiratory infections was concordant in 95.9% of cases for bronchitis with COPD, 85.6% for pharyngitis, 74.0% for pneumonia, and 90.0% for rhinosinusitis, regardless of comorbidities. The low concordance for pneumonia compared to other respiratory infections was due to the frequent prescription of amoxicillin clavulanate (15.0%), a non-recommended antibiotic. There was a high concordance in antibiotic choice for urinary tract infections (UTI) (94.9% for cystitis and 89.5% for pyelonephritis). For all infections, concordance decreased significantly when dosage was accounted for, reaching a low of 24.4% for rhinosinusitis in patients with one comorbidity. Conversely, concordance remained fairly high for patients with cystitis without a comorbidity, even when accounting for dosage (76.2%). Detailed results for adults can be found in Appendix 3 (Figs. 2, 3, 4 and 5).

**Table 3:** Concordance of antibiotic prescriptions and dosages with provincial guidelines, by number of comorbidities, in adults

Infection	Number of comorbidities	Prescriptions (*N*)	Concordant	Concordance Concordant + other*	Non-Concordant	Concordance when accounting for dosage
Bronchitis and COPD	0	--	--	--	--	--
	1	11 568	97.5%	1.0%	1.5%	52.6%
	2	7 245	96.9%	1.1%	2.0%	48.8%
	3+	21 269	94.7%	2.6%	2.7%	45.7%
	Total	40 082	95.9%	1.9%	2.2%	48.3%
Pharyngitis	0	21 959	87.5%	0.7%	11.8%	62.6%
	1	8 614	85.1%	0.7%	14.3%	56.9%
	2	3 202	82.9%	0.8%	16.3%	52.5%
	3+	3 229	77.0%	0.7%	22.4%	47.1%
	Total	37 004	85.6%	0.7%	13.7%	59.0%
Pneumonias	0	9 647	80.3%	0.5%	19.2%	50.9%
	1	9 353	77.0%	0.5%	22.4%	47.1%
	2	6 398	75.8%	0.8%	23.4%	44.8%
	3+	21 843	69.3%	1.2%	29.5%	35.4%
	Total	47 241	74.0%	0.9%	25.2%	42.2%
Rhinosinusitis	0	27 886	91.2%	0.5%	8.3%	25.2%
	1	18 552	89.9%	0.6%	9.6%	24.4%
	2	8 763	89.4%	0.7%	9.9%	25.8%
	3+	10 617	87.8%	0.9%	11.2%	25.3%
	Total	65 818	90.0%	0.6%	9.3%	25.1%
Cystitis	0	45 884	95.9%	1.3%	2.8%	76.2%
	1	32 881	95.7%	1.1%	3.3%	73.3%
	2	17 944	95.0%	1.1%	3.9%	70.9%
	3+	37 675	93.0%	1.3%	5.7%	62.7%
	Total	134 384	94.9%	1.2%	3.9%	71.0%
Pyelonephritis	0	6 277	91.4%	2.5%	6.1%	53.9%
	1	4 153	90.4%	3.0%	6.5%	52.0%
	2	2 399	90.0%	2.3%	7.7%	49.1%
	3+	6 173	86.9%	3.0%	10.1%	43.4%
	Total	19 002	89.5%	2.8%	7.7%	49.4%
URTI	0	12 211	0.0%	0.0%	100.0%	0.0%
	1	8 247	0.0%	0.0%	100.0%	0.0%
	2	4 253	0.0%	0.0%	100.0%	0.0%
	3+	6 329	0.0%	0.0%	100.0%	0.0%
	Total	31 040	0.0%	0.0%	100.0%	0.0%

Note: COPD = Chronic obstructive pulmonary disease, URTI = Upper respiratory tract infection, Provincial guidelines from 2016–2017, April 2018 to March 2022

*Concordant + other: when at least one recommended antibiotic is included in a combination of antibiotics, meaning more than one antibiotic is prescribed on the same day for the same infection

As URTIs were considered viral infections, the 31,040 prescriptions associated were considered non-concordant

Regression results for the pandemic period and the number of comorbidities are presented in Tables 4 and 5. For infections presented in Table 4, the analysis of the interaction between the two main variables was not statistically significant so only the main variables are presented. For bronchitis with COPD, one of the interaction terms was significant, so this infection is presented separately in Table 5. Similarly to what has been observed in children, adjusting for social and material deprivation indexes had only a minor impact on our regression results (Tables 4 and 5).

**Table 4:** Multivariate analysis of the impact of comorbidities and pandemic periods on concordance, in adults

Adults	Pneumonias		Pharyngitis		Rhinosinusitis		Cystitis		Pyelonephritis	
Variable	Crude RR (95% CI)	Adjusted RR* (95% CI)	Crude RR (95% CI)	Adjusted RR* (95% CI)	Crude RR (95% CI)	Adjusted RR* (95% CI)	Crude RR (95% CI)	Adjusted RR* (95% CI)	Crude RR (95% CI)	Adjusted RR* (95% CI)
**Period**										
Pre-pandemic period (April 2018 to March 2020)	Ref.	Ref.	Ref.	Ref.	Ref.	Ref.	Ref.	Ref.	Ref.	Ref.
Pandemic period (April 2020 to July 2021)	0.89 (0.86–0.92)	0.90 (0.86–0.93)	1.01 (0.98–1.03)	1.01 (0.99–1.04)	1.10 (1.06–1.15)	1.10 (1.06–1.15)	1.05 (1.04–1.06)	1.05 (1.05–1.06)	1.05 (1.02–1.09)	1.05 (1.02–1.08)
Post-pandemic period (August 2021 to March 2022)	1.02 (0.99–1.06)	1.02 (0.99–1.06)	1.05 (1.02–1.08)	1.05 (1.02–1.08)	1.32 (1.28–1.37)	1.32 (1.28–1.37)	1.07 (1.06–1.08)	1.08 (1.07–1.09)	1.13 (1.09–1.18)	1.12 (1.08–1.17)
**Number of comorbidities**										
0	Ref.	Ref.	Ref.	Ref.	Ref.	Ref.	Ref.	Ref.	Ref.	Ref.
1	0.93 (0.90–0.95)	0.94 (0.91–0.97)	0.91 (0.89–0.93)	0.95 (0.93–0.97)	0.97 (0.94-1.00)	0.97 (0.94-1.00)	0.96 (0.96–0.97)	0.99 (0.98–0.99)	0.97 (0.94–1.01)	0.97 (0.94–1.01)
2	0.88 (0.85–0.91)	0.91 (0.88–0.94)	0.84 (0.81–0.87)	0.92 (0.88–0.95)	1.02 (0.98–1.06)	1.02 (0.98–1.06)	0.93 (0.92–0.94)	0.97 (0.96–0.98)	0.91 (0.87–0.96)	0.91 (0.86–0.95)
3+	0.70 (0.68–0.72)	0.74 (0.72–0.76)	0.73 (0.67–0.79)	0.88 (0.85–0.92)	1.00 (0.97–1.04)	1.01 (0.97–1.05)	0.83 (0.82–0.83)	0.89 (0.88–0.90)	0.81 (0.78–0.84)	0.80 (0.77–0.84)

Note: CI = Confidence interval, RR = Relative risk, Ref = Reference category, Provincial guidelines from 2016–2017, April 2018 to March 2022. Both antibiotic choices and total dosages are included in the concordance definition. Since interaction terms were not statistically significant for these infections, we presented only the individual effects of each variable

*The adjusted models are adjusted for sex, age group, area of residence, social and material deprivation indexes

**Table 5:** Multivariate analysis of the combined impact of comorbidities and pandemic periods on concordance, for bronchitis

Adults	Bronchitis and COPD	
Variable	Crude RR (95% CI)	Adjusted RR (95% CI)
**Period among one comorbidity**		
Pre-pandemic period (April 2018 to March 2020)	Ref.	Ref.
Pandemic period (April 2020 to July 2021)	0.98 (0.94–1.01)	0.98 (0.94–1.01)
Post-pandemic period (August 2021 to March 2022)	1.11 (1.08–1.14)	1.11 (1.08–1.14)
**Number of comorbidities**		
1	Ref.	Ref.
2	0.93 (0.90–0.96)	0.93 (0.90–0.96)
3+	0.87 (0.85–0.89)	0.87 (0.85–0.89)
Interaction term (Comorbidity group * period)		
1 Comorbidity * Pre-pandemic period	Ref.	Ref.
2 Comorbidities * Pandemic period	0.95 (0.86–1.05)	0.95 (0.86–1.05)
3 + Comorbidities * Pandemic period	0.97 (0.90–1.05)	0.97 (0.90–1.05)
2 Comorbidities * Post-pandemic period	0.96 (0.89–1.05)	0.96 (0.89–1.05)
3 + Comorbidities * Post-pandemic period	0.90 (0.85–0.96)	0.90 (0.85–0.96)

Note: CI = Confidence interval, COPD = Chronic obstructive pulmonary disease, RR = Relative risk, Ref = Reference category, Provincial guidelines from 2016–2017, April 2018 to March 2022. Both antibiotic choices and total dosages are included in the concordance definition. Since interaction terms were statistically significant for bronchitis with COPD, we presented the interaction terms instead of the individual effects only. Only adults of 35 years of age or more are included in the bronchitis with COPD dataset

*The adjusted models are adjusted for sex, age group, area of residence, social and material deprivation indexes

In relation to comorbidities, we observed a significant decrease in total dosage concordance as the number of comorbidities increased, except for rhinosinusitis which showed no difference across all comorbidity groups. Adults with three or more comorbidities diagnosed with pneumonia and pyelonephritis showed the lowest drop in concordance (0.74, 0.72–0.76 for pneumonia and 0.80, 0.77–0.84 for pyelonephritis). Pandemic periods showed different tendencies depending on infection types. For pneumonia, a 10% decrease in concordance was observed during the pandemic period (0.90, 0.86–0.93). For other infections, regression results show gradual improvements in concordance through time, rather than evidence of a decrease during the pandemic. As for bronchitis with COPD, while the post-pandemic period was generally associated with an increase in concordance (1.11, 1.08–1.14), this was not observed in people with three comorbidities or more, given the statistically significant interaction term (0.90, 0.85–0.96).

Sensitivity analyses did not change the main conclusions (Appendix 4: Tables S2 and S3).

## Discussion

Between April 2018 and March 2022, antibiotic prescriptions for both children and adults under public drug insurance showed varying levels of concordance to provincial guidelines. In children, concordance was consistently high, exceeding 90% for all major infections and comorbidity groups, with slight decreases observed for pharyngitis and pneumonia in children with comorbidities. In adults, compliance varied significantly by infection type, reaching high rates for bronchitis with COPD (95.9%) but lower rates for pneumonia (74.0%) due to frequent use of non-recommended amoxicillin clavulanate. Dosage was notably less concordant for most infections, particularly in patients with multiple comorbidities, ranging from 24.4% for rhinosinusitis with one comorbidity to 76.2% for cystitis without any comorbidity. Rhinosinusitis had the lowest total dosage concordance of all infection types. While antibiotic prescribing trends changed with pandemic periods, we cannot attribute these changes to the COVID-19 pandemic.

In children, the statistically significant relative difference in proportions (difference of concordance) for pharyngitis (1.8%) and pneumonias (1.3%) in children with comorbidities have limited clinical and public health implications. Although small percentage variations can translate into thousands of cases in population-based studies, they do not necessarily justify targeted clinical interventions. Given the already high baseline concordance rates exceeding 90%, these slight variations are unlikely to meaningfully impact treatment outcomes in pediatric patients. The observed decline in concordance for otitis during the pandemic and the lower adherence for pharyngitis in the post-pandemic period may reflect transient changes in prescribing behaviors rather than systemic issues requiring intervention. Moreover, the pandemic period did not lead to major shifts in prescribing trends that would warrant further public health interventions.

In adults, the substantial variation in concordance by infection type and the impact of dosage on adherence highlight potential areas for intervention, but there is no evidence that the pandemic affected concordance systematically. The delayed adoption of the 2016–2017 guideline updates for rhinosinusitis, which halved recommended antibiotic dosages, likely contributed to the initially low concordance, which gradually improved over time, with a subsequent 10% increase in concordance across all comorbidity groups between 2018 and 2022. In a previous study using the same methodology and in the same population (years 2010–2017) to measure concordance with guidelines, most non-concordant dosages were higher than recommended [31]. Concordance increased during the pandemic and post-pandemic periods for most infections, suggesting a gradual adoption of the 2016–2017 guidelines but no clear negative impact of the pandemic. While this is good news, dosage adherence remained a concern, particularly for infections in patients with multiple comorbidities. Of note, deviations from guidelines are not necessarily inappropriate, especially in patients with multiple comorbidities, for whom drug interaction might need to be avoided. The absence of a significant interaction between comorbidities and pandemic periods in most cases suggests that underlying conditions did not substantially alter antibiotic prescribing trends during this time, except for bronchitis in patients with COPD, with interaction detected during the post-pandemic period. Clinically, this suggests that the presence of multiple comorbidities may not have significantly influenced the management of infections during the pandemic. Finally, sensitivity analysis (where antibiotic combinations were considered concordant if at least one antibiotic was concordant) did not alter the overall conclusions of the study.

A decrease in concordance with the presence of comorbidities was also reported in other studies from Quebec [31, 32], which showed a 12% drop in concordance percentage for UTIs in people with 4 or more chronic conditions between 2002 and 2017 [31, 32]. The authors also observed a slight decrease in concordance with antibiotic prescription guidelines in presence of specific chronic conditions such as diabetes, mental health disorders, and cardiovascular and respiratory diseases [32]. Fortin et al. highlighted that the QICDSS relies on sensitive algorithms to detect chronic conditions but does not distinguish early and advanced stages [32]. This could weaken the association between non-concordance and chronic conditions. Other studies further illustrate the negative association between comorbidities and concordance with clinical guidelines. A British study observed a positive association between adults with comorbidities and inappropriate antibiotics prescriptions for URTIs, lower respiratory tract infections (LRTIs) and UTIs [33]. Furthermore, Saatchi et al. conducted a Canadian retrospective analysis on antibiotic prescription quality in older adults with pneumonia in the community, comparing adherence to the Infectious Diseases Society of America (IDSA) guidelines [34]. They found that fewer than 2% of prescriptions perfectly matched the recommendations for antibiotic selection, dosage, and treatment duration. However, 60–75% of prescriptions were classified as “clinically appropriate,” often justified by factors such as patient comorbidities. Given that nearly half of the older population had a Charlson Comorbidity Index (CCI) score of at least one, the authors argued that deviations from guidelines could be reasonable for older or multimorbid patients. These findings could explain our results, since there is an over-representation of older adults in the public drug insurance plan in Quebec and that older people have more comorbidities [23, 35]. Deviation from clinical guidelines for people with comorbidities could be explained by polypharmacy, which is the simultaneous use of multiple medications [36, 37]. Prescribing non recommended antibiotics may become necessary to avoid drug interactions, an issue that was documented over the years and could therefore explain part of the discordance with clinical guidelines for antibiotic prescriptions in individuals with multimorbidity [32, 38, 39].

The impact of the COVID-19 pandemic on rates of antibiotic use has been documented elsewhere [2, 16, 40]. It is also known that the pandemic disrupted the healthcare system [13, 41]. To our knowledge, the impact of the COVID-19 pandemic and of the number of comorbidities on guidelines concordance in the community had not been documented in Quebec or Canada. The trends we observed tended to differ according to the type of infection during the pandemic. We observed an increase of the concordance during the pandemic for rhinosinusitis, cystitis and pyelonephritis. Those results are similar to results reported by another Quebec study team on the WHO AWaRe recommendations [16]. The authors observed an increase in the proportion of prescriptions belonging to the “Access” category, but did not have information on treatment indications. Our study goes a step further and analysed the concordance with local guidelines per type of infections. However, since there was no statistically significant interaction except for bronchitis, and the effect of pandemic periods independently of the number of comorbidities varied according to the type of infection, it was difficult to determine whether the COVID-19 pandemic had an impact on prescription concordance. A major change during the pandemic was the increased popularity of telehealth consultations [14]. This could have changed prescribing habits for primary care physicians, as diagnosis of patients in a virtual setting can be challenging. However, studies seem to disagree on the effect telehealth had on antibiotic usage. Ray et al. compared prescriptions trends across three types of consultations: telemedicine, emergency care and primary care [42]. Telemedicine showed the highest prescription rate (52% compared to 42% in emergency care and 31% in primary care) and the lowest guideline concordance (59% compared to 67% in emergency care and 78% in primary care). An American study also showed that virtual consultations were associated with higher antibiotic prescription rates (58% vs. 43% in-person) [43]. In contrast, the authors of another American study observed no statistically significant difference between the two types of consultations (28% in-person and 29% via telemedicine) [44]. Unfortunately, we were unable to verify this possible association in our dataset.

### Strengths and limitations

To our knowledge, this is the first study to evaluate the combined effect of the number of comorbidities and the COVID-19 pandemic on adherence to local clinical guidelines. Moreover, this study benefited from large sample sizes, especially in analyzing infections among adults. Additionally, previous studies have shown the quality and validity of both the health administrative data used and the algorithms applied to identify comorbidities [25, 29, 45, 46]. It also has been demonstrated that QICDSS databases can be used for surveillance of antibiotic utilization [47]. Finally, using a medico-administrative database eliminates the need for participant recruitment, saving considerable time, scarce resources and costs.

Our definition of concordance was driven by the available data and covers only part of the larger concept of appropriate treatment. Because diagnosis is not mandatory in physician claims, we were unable to identify all infections covered by guidelines and could therefore not consider non-prescription (usually good practice) in our measurements. Key clinical information was either completely absent (allergies, stage of chronic diseases) or had to be imputed (diagnosis) or approximated (treatment failure, complex infections); we addressed this by restricting our analysis to simple scenarios to favor correct imputation or approximation and we treated as concordant any regimen recommended in the guidelines, which may lead to higher estimates of concordance. Finally, deviations from guidelines may reflect appropriate clinical decisions, especially in patients with multiple comorbidities; the differences observed in concordance by number of comorbidities may be justified, at least partly. In summary, concordance with the guidelines whenever a prescription with a diagnosis is delivered remains important information. It can also be followed in time, as to measure the impact of the pandemic, so while the definition has limitations, the measure and the way we use it were still relevant for our purposes.”

There are other limitations to this study. To begin with, individuals 65 years and over and people with lower socio-economic status younger than 65 years are overrepresented in the public drug insurance plan, as mentioned in the description of the study population [23]. Fortunately, material and social deprivation indexes had little impact on our results. Therefore, the impact of this limitation, if present, should be limited. Certain groups were excluded from the study, such as those in long-term care, since their medication is provided by the establishment’s pharmacies, as well as individuals from Nunavik and Terres-Cries-de-la-Baie-James due to incomplete data on prescriptions [25]. Moreover, we did not have access to patients’ medical records but rather to physician billing data. This limitation is partly addressed by Cadieux et al., whose study demonstrated the adequate sensitivity and specificity of public health insurance plan medical data [45]. Another limitation is the potential for misclassification, which could arise if a patient refrains from seeking healthcare services. In such cases, any undiagnosed comorbidities would not be captured in the QICDSS. As a result, individuals with undiagnosed comorbidities may have been inaccurately categorized as having no comorbidities, potentially leading to an underestimation of the relationship between antibiotic use and the number of comorbidities. Additionally, the identification of chronic diseases was conducted using complex algorithms, which may include comorbidities not linked to antibiotic use or conditions in early stages. This could potentially lead to an underestimation of the impact of more advanced comorbidities on antibiotic prescription concordance with guidelines [32]. Finally, we could not investigate the potential impact of telemedicine on concordance of antibiotic prescriptions with local guidelines, which could be interesting for a better understanding of the impact of this practice.

## Conclusion

Antibiotic choice was highly concordant with guidelines for children, especially for otitis and pneumonia. Adult prescriptions also showed high concordance of antibiotic choice for bronchitis with COPD and cystitis. In adults, when total dosage was accounted for, the concordance dropped significantly, especially for rhinosinusitis. Adherence to clinical guidelines decreased as the number of comorbidities increased. As for the pandemic periods, results varied from an infection type to another and could reflect slowly increasing adoption of recommendations rather than an influence of the pandemic itself. The pandemic itself seemed to have little impact on the association between the number of comorbidities and concordance. To address AMR and enhance antibiotic adherence, educational training could be offered to prescribers and clinicians, focusing on AMR, particularly in adult infections. Public awareness campaigns could emphasize responsible antibiotic use, while clinical guidelines may be disseminated via online platforms, journals, workshops, and email newsletters. Ongoing efforts are essential to assess the long-term impact on antibiotic resistance and to refine prescribing practices in an evolving healthcare environment.

## Supplementary Information

Below is the link to the electronic supplementary material.

Supplementary Material 1

## Data Availability

Data used for this study cannot be shared, however, questions can be addressed to the corresponding author.

## Abbreviations

AMR: Antimicrobial resistance
AMU: Antimicrobial use
ATC: Anatomical Therapeutic Chemical
CAI: Commission d’accès à l’information du Québec
CCI: Charlson comorbidity index
CI: Confidence interval
COPD: Chronic obstructive pulmonary disease
FIPA: Fichier d’inscription des personnes assurées
GLASS: Global Antimicrobial Resistance and Use Surveillance System
ICD-9: International Classification of Diseases, Ninth Revision
ICD-10: International Classification of Diseases, Tenth Revision
IDSA: Infectious diseases society of America
INESSS: Institut national d’excellence en santé et services sociaux
INSPQ: Institut national de santé publique du Québec
LRTI: Lower respiratory tract infection
MED-ECHO: Maintenance et exploitation des données pour l’étude de la clientèle hospitalière
QIC: Quasi-likelihood under the independence model criterion
QICDSS: Quebec Integrated Chronic Disease Surveillance System
RAMQ: Régie de l’assurance maladie du Québec
RPAM: Régime public d’assurance médicaments
UTI: Urinary tract infection
URTI: Upper respiratory tract infection
WHO: World Health Organization
