# Supplementary material for: Joint impact of the number of comorbidities and the COVID-19 pandemic on the concordance of antibiotic prescriptions with clinical guidelines, in the community
Source: BMC Infect Dis. 2026 Jul 8;26:1762. doi: 10.1186/s12879-026-13948-6 (PMC13632065; doi:10.1186/s12879-026-13948-6)
Supplement: Supplementary file 1 [file 12879_2026_13948_MOESM1_ESM.docx]

**Appendix 1: Table of diagnostic codes defining the different types of infection**

| **Infection type** | **Infection** | **ICD-9 codes** | **ICD-10 codes** |
| --- | --- | --- | --- |
| Respiratory | Bronchitis and COPD exacerbation | 466 466.0 466.1 466.9  490 490.9 | J20.1 J20.6 J20.9 J21.9  J40 |
| Respiratory | Otitis media | 381.0 381.4  382.0 382.4 382.9 | H65.0 H65.1 H65.9  H66.0 H66.4 H66.9 |
| Respiratory | Pharyngitis | 034 034.0 034.1  462  462.9  463 463.9 | A38  J02.0 J02.8 J02.9  J03.0 J03.8 J03.9 |
| Respiratory | Pneumonia | 481 481.9  482 482.0 482.1 482.2 482.3 482.4 482.8 482.9  485 485.9  486 486.9 | J13  J14  J15.1 J15.8 J15.9  J18.0 J18.1 J18.8 J18.9 |
| Respiratory | Sinusitis | 461 461.0 461.1 461.2 461.3 461.8 461.9 | J01.0 J01.1 J01.3 J01.4 J01.8 J01.9 |
| Respiratory | URTI | 460 460.9  464 464.0 464.1 464.2 464.3 464.4 464.9  465 465.0 465.8 465.9 | J00  J04.0 J04.1 J04.2  J05.0 J05.1  J06.0 J06.8 J06.9 |
| Urinary | Cystitis | 595 595.0 595.1 595.2 595.3 595.4 595.8 595.9 | N30.0 N30.1 N30.2 N30.3 N30.4 N30.8 N30.9 |
| Urinary | Pyelonephritis | 590 590.1 590.2 590.3 590.8 590.9 | N10  N11.0 N11.1  N13.6 |

**Appendix 2: Summary of the 2016-2017 provincial clinical guidelines**

| **Infection** | **Age group** | **Antibiotic 1** | **Antibiotic 2** | **Minimum total dosage 1** | **Maximum total dosage 1** | **Minimum total dosage 2** | **Maximum total dosage 2** |
| --- | --- | --- | --- | --- | --- | --- | --- |
| Pneumonia | adult | Clarithromycin |  | 7000 | 7000 |  |  |
| Pneumonia | adult | Azithromycin |  | 1500 | 1500 |  |  |
| Pneumonia | adult | Doxycycline |  | 1400 | 1400 |  |  |
| Pneumonia | adult | Amoxicillin |  | 21000 | 21000 |  |  |
| Pneumonia | adult | Amoxicillin | Clarithromycin | 21000 | 21000 | 7000 | 7000 |
| Pneumonia | adult | Amoxicillin | Azithromycin | 21000 | 21000 | 1500 | 1500 |
| Pneumonia | adult | Amoxicillin | Doxycycline | 21000 | 21000 | 1400 | 1400 |
| Pneumonia | adult | Amoxi-clav | Clarithromycin | 14000 | 14000 | 7000 | 7000 |
| Pneumonia | adult | Amoxi-clav | Azithromycin | 14000 | 14000 | 1500 | 1500 |
| Pneumonia | adult | Amoxi-clav | Doxycycline | 14000 | 14000 | 1400 | 1400 |
| Pneumonia | adult | Levofloxacin |  | 3500 | 7500 |  |  |
| Pneumonia | adult | Moxifloxacin |  | 2800 | 2800 |  |  |
| Pneumonia | adult | Cefuroxime | Clarithromycin | 7000 | 7000 | 7000 | 7000 |
| Pneumonia | adult | Cefuroxime | Azithromycin | 7000 | 7000 | 1500 | 1500 |
| Pneumonia | adult | Cefuroxime | Doxycycline | 7000 | 7000 | 1400 | 1400 |
| Pneumonia | adult | Cefadroxil | Clarithromycin | 7000 | 7000 | 7000 | 7000 |
| Pneumonia | adult | Cefadroxil | Azithromycin | 7000 | 7000 | 1500 | 1500 |
| Pneumonia | adult | Cefadroxil | Doxycycline | 7000 | 7000 | 1400 | 1400 |
| Pneumonia | adult | Cefprozil | Clarithromycin | 7000 | 7000 | 7000 | 7000 |
| Pneumonia | adult | Cefprozil | Azithromycin | 7000 | 7000 | 1500 | 1500 |
| Pneumonia | adult | Cefprozil | Doxycycline | 7000 | 7000 | 1400 | 1400 |
| Sinusitis | adult | Amoxicillin |  | 7500 | 7500 |  |  |
| Sinusitis | adult | Amoxi-clav |  | 13125 | 14000 |  |  |
| Sinusitis | adult | Cefixime |  | 4000 | 4000 |  |  |
| Sinusitis | adult | Cefuroxime |  | 3500 | 3500 |  |  |
| Sinusitis | adult | Clarithromycin |  | 7000 | 7000 |  |  |
| Sinusitis | adult | Doxycycline |  | 2000 | 2000 |  |  |
| Sinusitis | adult | Trimethoprim-sulfamethoxazole |  | 13440 | 13440 |  |  |
| Sinusitis | adult | Amoxi-clav |  | 13125 | 14000 |  |  |
| Sinusitis | adult | Levofloxacin |  | 5000 | 5000 |  |  |
| Sinusitis | adult | Moxifloxacin |  | 2000 | 2000 |  |  |
| Sinusitis | child | Amoxicillin |  |  | 56000 |  |  |
| Sinusitis | child | Amoxicillin | Amoxi-clav |  | 21000 |  | 21000 |
| Sinusitis | child | Amoxi-clav |  |  | 42000 |  |  |
| Sinusitis | child | Cefprozil |  |  | 14000 |  |  |
| Sinusitis | child | Cefuroxime |  |  | 14000 |  |  |
| Sinusitis | child | Clarithromycin |  |  | 14000 |  |  |
| Pharyngitis | child | Phenoxymethylpenicillin |  |  | 12000 |  |  |
| Pharyngitis | child | Amoxicillin |  |  | 10000 |  |  |
| Pharyngitis | child | Azithromycin |  |  | 2500 |  |  |
| Pharyngitis | child | Cefalexin |  |  | 10000 |  |  |
| Pharyngitis | child | Clarithromycin |  |  | 5000 |  |  |
| Pharyngitis | adult | Phenoxymethylpenicillin |  | 12000 | 12000 |  |  |
| Pharyngitis | adult | Amoxicillin |  | 10000 | 10000 |  |  |
| Pharyngitis | adult | Azithromycin |  | 1500 | 1500 |  |  |
| Pharyngitis | adult | Cefadroxil |  | 10000 | 10000 |  |  |
| Pharyngitis | adult | Cefalexin |  | 10000 | 10000 |  |  |
| Pharyngitis | adult | Clarithromycin |  | 5000 | 5000 |  |  |
| Pharyngitis | adult | Clindamycin |  | 9000 | 9000 |  |  |
| Pneumonia | child | Amoxicillin |  |  | 30000 |  |  |
| Pneumonia | child | Amoxi-clav |  |  | 30000 |  |  |
| Pneumonia | child | Amoxicillin | Amoxi-clav |  | 15000 |  | 15000 |
| Pneumonia | child | Cefuroxime |  |  | 10000 |  |  |
| Pneumonia | child | Cefprozil |  |  | 10000 |  |  |
| Pneumonia | child | Azithromycin |  |  | 1500 |  |  |
| Pneumonia | child | Clarithromycin |  |  | 10000 |  |  |
| Otitis | child | Amoxicillin |  |  |  |  |  |
| Otitis | child | Amoxi-clav |  |  |  |  |  |
| Otitis | child | Amoxicill | Amoxi-clav |  |  |  |  |
| Otitis | child | Ceftriaxone |  |  |  |  |  |
| Otitis | child | Cefprozil |  |  |  |  |  |
| Otitis | child | Cefuroxime |  |  |  |  |  |
| Otitis | child | Clarithromycin |  |  |  |  |  |
| Otitis | child | Azithromycin |  |  |  |  |  |
| Cystitis in women | adult | Nitrofurantoin |  | 1000 | 2000 |  |  |
| Cystitis in women | adult | Fosfomycin |  | 3000 | 9000 |  |  |
| Cystitis in women | adult | Trimethoprim-sulfamethoxazole |  | 5280 | 19200 |  |  |
| Cystitis in women | adult | Amoxi-clav |  | 10000 | 20000 |  |  |
| Cystitis in women | adult | Trimethoprim |  | 600 | 600 |  |  |
| Cystitis in women | adult | Cefadroxil |  | 5000 | 10000 |  |  |
| Cystitis in women | adult | Cefalexin |  | 14000 | 14000 |  |  |
| Cystitis in women | adult | Cefixime |  | 2000 | 4000 |  |  |
| Cystitis in women | adult | Ciprofloxacin |  | 1500 | 3500 |  |  |
| Cystitis in women | adult | Levofloxacin |  | 750 | 1750 |  |  |
| Pyelonephritis or cystitis in men | adult | Ciprofloxacin |  | 7000 | 10000 |  |  |
| Pyelonephritis or cystitis in men | adult | Levofloxacin |  | 3500 | 7000 |  |  |
| Pyelonephritis or cystitis in men | adult | Ciprofloxacin |  | 10000 | 14000 |  |  |
| Pyelonephritis or cystitis in men | adult | Levofloxacin |  | 5000 | 7000 |  |  |
| Pyelonephritis or cystitis in men | adult | Trimethoprim-sulfamethoxazole |  | 13440 | 26880 |  |  |
| Pyelonephritis or cystitis in men | adult | Amoxi-clav |  | 20000 | 28000 |  |  |
| Pyelonephritis or cystitis in men | adult | Cefadroxil |  | 10000 | 14000 |  |  |
| Pyelonephritis or cystitis in men | adult | Cefixime |  | 4000 | 5600 |  |  |
| Pyelonephritis or cystitis in men | adult | Cefalexin |  | 20000 | 28000 |  |  |
| Bronchitis with COPD | adult | Azithromycin |  | 1500 | 1500 |  |  |
| Bronchitis with COPD | adult | Clarithromycin |  | 5000 | 7000 |  |  |
| Bronchitis with COPD | adult | Doxycycline |  | 1400 | 1400 |  |  |
| Bronchitis with COPD | adult | Amoxicillin |  | 15000 | 21000 |  |  |
| Bronchitis with COPD | adult | Azithromycin |  | 1500 | 1500 |  |  |
| Bronchitis with COPD | adult | Clarithromycin |  | 5000 | 7000 |  |  |
| Bronchitis with COPD | adult | Cefuroxime |  | 5000 | 7000 |  |  |
| Bronchitis with COPD | adult | Doxycycline |  | 1000 | 1400 |  |  |
| Bronchitis with COPD | adult | Trimethoprim-sulfamethoxazole |  | 9600 | 13440 |  |  |
| Bronchitis with COPD | adult | Cefprozil |  | 5000 | 7000 |  |  |
| Bronchitis with COPD | adult | Amoxi-clav |  | 10000 | 14000 |  |  |
| Bronchitis with COPD | adult | Levofloxacin |  | 3750 | 5250 |  |  |
| Bronchitis with COPD | adult | Moxifloxacin |  | 2000 | 2000 |  |  |

**Appendix 3:**

Figure 1: Trends of concordance with guidelines according to the number of comorbidities, in children

Note: April 2018 to March 2022, provincial guidelines from 2016-2017.

Figure 2: Trends of concordance with guidelines according to number of comorbidities, for respiratory infections, in adults

Note: April 2018 to March 2022, provincial guidelines from 2016-2017. As per the infection definition, bronchitis with COPD does not have a zero-comorbidity category.

Figure 3: Trends of concordance with guidelines according to number of comorbidities, for urinary infections, in adults

Note: April 2018 to March 2022, provincial guidelines from 2016-2017.

Figure 4: Trends of concordance with guidelines when accounting for dosage according to number of comorbidities, for respiratory infections, in adults

Note: April 2018 to March 2022, provincial guidelines from 2016-2017. This is a descriptive analysis of antibiotic total dosages for respiratory infections in adults.

Figure 5: Trends of concordance with guidelines when accounting for dosage, according to number of comorbidities, for urinary infections, in adults

Note: April 2018 to March 2022, provincial guidelines from 2016-2017. This is a descriptive analysis of antibiotic total dosages for urinary tract infections in adults.

**Appendix 4:**

| **Children** | **Pneumonias***** | | **Pharyngitis** | | **Rhinosinusitis** | | **Otitis** | |
| --- | --- | --- | --- | --- | --- | --- | --- | --- |
| **Variable** | **Crude RR (95% CI)** | **Adjusted RR****  **(95% CI)** | **Crude RR (95% CI)** | **Adjusted RR****  **(95% CI)** | **Crude RR (95% CI)** | **Adjusted RR****  **(95% CI)** | **Crude RR (95% CI)** | **Adjusted RR****  **(95% CI)** |
| **Period** |  |  |  |  |  |  |  |  |
| Pre-pandemic period  (April 2018 to March 2020) | Ref. | Ref. | Ref. | Ref. | Ref. | Ref. | Ref. | Ref. |
| Pandemic period  (April 2020 to July 2021) | 0.988  (0.971-1.005) | 0.984  (0.966-1.002) | 1.002  (0.988-1.016) | 1.001  (0.995-1.023) | 0.972  (0.931-1.014) | 0.969  (0.923-1.012) | 0.995  (0.991-0.998) | 0.995  (0.991-0.998) |
| Post-pandemic period  (August 2021 to March 2022) | 1.001  (0.993-1.010) | 0.997  (0.986-1.006) | 0.978  (0.963-0.994) | 0.982  (0.967-0.998) | 0.994  (0.966-1.023) | 0.992  (0.964-1.021) | 0.998  (0.995-1.000) | 0.998  (0.995-1.000) |
|  |  |  |  |  |  |  |  |  |
| **Number of comorbidities** |  |  |  |  |  |  |  |  |
| 0 | Ref. | Ref. | Ref. | Ref. | Ref. | Ref. | Ref. | Ref. |
| 1+ | 0.988  (0.981-0.996) | 0.988  (0.980-0.996) | 0.981  (0.967-0.993) | 0.982  (0.970-0.994) | 0.993  (0.972-1.014) | 0.995  (0.974-1.016) | 0.998  (0.995-1.000) | 0.998  (0.995-1.001) |

**Table S1**: Sensitivity analysis of the impact of comorbidities and pandemic periods on concordance, in children

Note: CI = Confidence interval, RR = Relative risk, Ref = Reference category, April 2018 to March 2022, provincial guidelines from 2016-2017.

* Sensitivity analysis: the "concordant+other" category was included in the "Concordant" category.

**The adjusted models are adjusted for sex, age group, area of residence, social and material deprivation indexes.

*** In order to protect the identity of individuals in cells with small numbers, for pneumonias in children, the model was not adjusted for the social and material deprivation indexes and the 12-17 years old age group was included in the 5-11 years old age group.

**Table S2**: Sensitivity analysis of the impact of comorbidities and pandemic periods on concordance, in adults

| **Adults** | **Pneumonias** | | **Pharyngitis** | | **Rhinosinusitis** | | **Bronchitis and COPD** | |  |  |
| --- | --- | --- | --- | --- | --- | --- | --- | --- | --- | --- |
| **Variable** | **Crude RR**  **(95% CI)** | **Adjusted RR** (95% CI)** | **Crude RR**  **(95% CI)** | **Adjusted RR** (95% CI)** | **Crude RR**  **(95% CI)** | **Adjusted RR** (95% CI)** | **Crude RR**  **(95% CI)** | **Adjusted RR** (95% CI)** |  |  |
| **Period** |  |  |  |  |  |  |  |  |  |  |
| Pre-pandemic period  (April 2018 to March 2020) | Ref. | Ref. | Ref. | Ref. | Ref. | Ref. | Ref. | Ref. |  | |
| Pandemic period  (April 2020 to July 2021) | 0.93 (0.90-0.96) | 0.93 (0.90-0.96) | 1.01 (0.98-1.03) | 1.01 (0.99-1.04) | 1.10 (1.06-1.15) | 1.10 (1.06-1.15) | 0.99 (0.95-1.02) | 0.98 (0.95-1.01) |  | |
| Post-pandemic period  (August 2021 to March 2022) | 1.05 (1.02-1.07) | 1.05 (1.02-1.07) | 1.05 (1.02-1.08) | 1.05 (1.02-1.08) | 1.32 (1.28-1.37) | 1.32 (1.28-1.37) | 1.11 (1.08-1.14) | 1.11 (1.08-1.14) |  | |
|  |  |  |  |  |  |  |  |  |  | |
| **Number of comorbidities** |  |  |  |  |  |  |  |  |  | |
| 0 | Ref. | Ref. | Ref. | Ref. | Ref. | Ref. | -- | -- |  | |
| 1 | 0.94 (0.91-0.96) | 0.94 (0.92-0.97) | 0.91 (0.89-0.93) | 0.95 (0.93-0.97) | 0.97 (0.94-1.00) | 0.97 (0.94-1.00) | Ref. | Ref. |  | |
| 2 | 0.92 (0.89-0.94) | 0.93 (0.91-0.96) | 0.84 (0.82-0.87) | 0.92 (0.89-0.95) | 1.02 (0.98-1.07) | 1.03 (0.98-1.07) | 0.93 (0.90-0.96) | 0.92 (0.90-0.95) |  | |
| 3+ | 0.78 (0.76-0.80) | 0.80 (0.78-0.82) | 0.76 (0.73-0.79) | 0.88 (0.85-0.92) | 1.01 (0.97-1.05) | 1.01 (0.97-1.06) | 0.89 (0.87-0.91) | 0.89 (0.87-0.91) |  | |

Note: CI = Confidence interval, COPD = Chronic obstructive pulmonary disease, RR = Relative risk, Ref = Reference category, April 2018 to March 2022, provincial guidelines from 2016-2017. Only respiratory infections.

* Sensitivity analysis: the "concordant+other" category was included in the "Concordant" category.

**The adjusted models are adjusted for sex, age group, area of residence, social and material deprivation indexes.

**Table S3***:* Sensitivity analysis of the impact of comorbidities and pandemic periods on concordance, in adults

| **Adults** | **Cystitis** | | **Pyelonephritis** | |  |
| --- | --- | --- | --- | --- | --- |
|  |  |  |  |  |  |
| **Variable** | **Crude RR (95% CI)** | **Adjusted RR** (95% CI)** | **Crude RR (95% CI)** | **Adjusted RR** (95% CI)** |  |
| **Period** |  |  |  |  |  |
| Pre-pandemic period  April 2018 to March 2020) | Ref. | Ref. | Ref. | Ref. |  |
| Pandemic period  (April 2020 to July 2021) | 1.05 (1.04-1.06) | 1.05 (1.05-1.06) | 1.06 (1.03-1.10) | 1.06 (1.03-1.09) |  |
| Post-pandemic period  (August 2021 to March 2022) | 1.06 (1.05-1.07) | 1.07 (1.06-1.08) | 1.14 (1.09-1.18) | 1.13 (1.09-1.17) |  |
|  |  |  |  |  |  |
| **Number of comorbidities** |  |  |  |  |  |
| 0 | Ref. | Ref. | Ref. | Ref. |  |
| 1 | 0.96 (0.95-0.97) | 0.99 (0.98-0.99) | 0.97 (0.94-1.01) | 0.98 (0.94-1.01) |  |
| 2 | 0.93 (0.92-0.94) | 0.97 (0.96-0.98) | 0.91 (0.87-0.95) | 0.90 (0.86-0.94) |  |
| 3+ | 0.83 (0.82-0.83) | 0.89 (0.88-0.90) | 0.81 (0.79-0.84) | 0.81 (0.78-0.84) |  |

Note: CI = Confidence interval, RR = Relative risk, Ref = Reference category, April 2018 to March 2022, provincial guidelines from 2016-2017. Only respiratory infections. Only urinary tract infections.

* Sensitivity analysis: the "concordant+other" category was included in the "Concordant" category.

**The adjusted models are adjusted for sex, age group, area of residence, social and material deprivation indexes.
